# Gene Expression Alterations in Peripheral Blood Mononuclear Cells and Cartilage Explants from End-Stage Rheumatoid Arthritis Patients in Response to Taurine: A Pilot Exploratory Study

**DOI:** 10.3390/life16050791

**Published:** 2026-05-09

**Authors:** Elena Tchetina, Irina Kushnareva, Ekaterina Anisimova, Angele Vienozinskaite, Oksana Plastinina, Maksim Makarov, Aleksandr Lila

**Affiliations:** 1 Immunology and Molecular Biology Department, Nasonova Research Institute of Rheumatology, Moscow 115522, Russia; katia.an2016@yandex.ru (E.A.); angelina_vienozinskaite@hotmail.com (A.V.); 2 Surgery Department, Nasonova Research Institute of Rheumatology, Moscow 115522, Russia; dr.kushnareva@yandex.ru (I.K.); oksanaplastinina@rambler.ru (O.P.); ortopedniir@mail.ru (M.M.); 3 Osteoartritis Laboratory, Nasonova Research Institute of Rheumatology, Moscow 115522, Russia; amlila@mail.ru

**Keywords:** rheumatoid arthritis, gene expression, taurine, PBMCs, articular cartilage

## Abstract

The aim of this preliminary study was to determine the effect of taurine on the expression of genes involved in glycolysis, oxidative phosphorylation, inflammation, autophagy, and regenerative activity in cultured peripheral blood mononuclear cells (PBMCs) and articular cartilage explants from patients with end-stage rheumatoid arthritis (RA). PBMCs and knee articular cartilage were obtained from 20 patients with RA (3 men and 17 women) aged 62.2 ± 10.9 years, with a mean disease duration of 17.5 years (range: 2–43), prior to arthroplasty. PBMCs and cartilage explants were cultured in the presence of 50 µM taurine. Gene expression was determined using real-time reverse transcriptase polymerase chain reaction (RT-PCR). Protein expression of the examined genes in PBMCs was quantified using ELISA. In the presence of 50 µM taurine PBMCs from patients with RA demonstrated a significant increase in the expression of genes encoding pyruvate kinase (PKM2), succinate dehydrogenase (SDHB), uncoupler of oxidation and phosphorylation (UCP2), ATP synthase (ATP5B), and unc-51-like kinase 1 (ULK1). At the same time a significant decrease in tumor necrosis factor (TNF)α and interleukin (IL)-1β expression was noted. In cartilage explants, taurine upregulated SDHB, UCP2, ULK1, and type 2 collagen gene (COL2A1), and decreased TNFα expression. We concluded that, under in vitro conditions, taurine can influence the expression of genes involved in glycolysis, oxidative phosphorylation, inflammation, autophagy, and regenerative processes in PBMCs and articular chondrocytes from patients with end-stage RA.

## 1. Introduction

Rheumatoid arthritis (RA) is a chronic autoimmune rheumatic disease of unknown etiology. It is characterized by erosive polyarthritis that can lead to joint destruction and systemic inflammatory lesions of internal organs [1]. The prevalence of RA in the adult population ranges from 0.5% to 1% [2]. Furthermore, it has been noted that without treatment, 50% of patients diagnosed with RA develop disability within two years [3]. Currently, RA therapy primarily involves NSAIDs, glucocorticoids, biologics, and JAK kinase inhibitors [4]. However, some patients do not respond to these treatments, experiencing persistent joint pain and swelling, which indicates limited efficacy in slowing disease progression or preventing tissue destruction [5].

Pathological processes in RA involve both inflammation and the disruption of central metabolic pathways, which affect the expression of genes regulating energy metabolism [6]. Evidence suggests that, due to the high protein biosynthetic activity required for cell proliferation and pro-inflammatory cytokine synthesis, glucose is redirected into the pentose phosphate pathway in lymphocytes of patients with RA. Consequently, less glucose is utilized for ATP production via glycolysis—a process mediated by key enzymes such as pyruvate kinase (PKM)—resulting in reduced glycolytic activity and diminished ATP generation [7]. Furthermore, decreased pyruvate concentrations impede the Krebs cycle, particularly affecting the expression of related enzymes such as succinate dehydrogenase (SDH). This subsequently leads to a reduction in ATP synthesis via the electron transport chain (ETC) and ATP synthase [8].

Furthermore, reduced succinate dehydrogenase activity leads to the accumulation of succinate, which may induce angiogenesis and promote immune cell infiltration into the synovial tissue of rheumatoid joints [9]. Additionally, the intracellular redistribution of AMP-activated protein kinase (AMPK) in lymphocytes from patients with RA is impaired; it fails to accumulate at the lysosomal membrane, thereby preventing it from exerting its regulatory functions [6]. This impairment results in heightened proliferative activity, an accumulation of intracellular metabolites, and defects in DNA repair within T lymphocytes, which weakens their ability to eliminate dysfunctional molecules through ULK1-mediated autophagy [10]. Consequently, restoring normal metabolism in T lymphocytes via cell reprogramming is essential for disease treatment [11]. Moreover, it has been demonstrated that clinical remission does not restore gene expression levels to those observed in healthy individuals. Instead, remission is associated with the upregulation of glycolysis-related pyruvate kinase M2 (PKM2) and Krebs cycle-related succinate dehydrogenase (SDHB) genes, as well as more efficient respiratory chain function, such as during tofacitinib therapy [12].

Given that anti-rheumatic drugs often cause adverse effects and frequently require repeated courses, there is a pressing need for additional therapeutic approaches [13]. In this context, exploring nutrients as potential supplements is promising, as they are generally non-toxic and may positively influence patient outcomes. One such nutrient is taurine. As an organic osmolyte, taurine regulates cell volume and serves as a substrate for bile synthesis. It is present in significant quantities in all eukaryotes, including humans, and is considered non-toxic [14]. Unlike most amino acids, taurine is not incorporated into proteins; it is partially synthesized from cysteine or obtained from dietary sources via taurine transporters [15]. Recently, interest in taurine as a dietary supplement has grown, bolstered by animal studies demonstrating that taurine supplementation can improve organ function in young subjects [16]. Furthermore, taurine deficiency may be a driver of aging, as supplementation has been shown to increase the healthy lifespan of animals [17]. Growing interest also surrounds the use of taurine to manage metabolic disorders affecting muscle, cardiac function, liver activity, and adipose tissue [18]. In humans, lower levels of taurine pathway metabolites have been associated with age-related diseases such as obesity, diabetes, and inflammation [17], while supplementation has proven effective in preventing these systemic pathologies [19]. Additionally, taurine may play a role in central nervous system development [20], and fluctuations in taurine levels and its transporters have been linked to cognitive impairments [21]. The antioxidant properties of taurine enhance mitochondrial function by stabilizing the electron transport chain and inhibiting the production of free radicals [22]. It has been proposed that when the electron transport chain is disrupted, electron donors accumulate and may be diverted to oxygen, thereby forming superoxide anions [23]. Increasing taurine concentration restores electron transport chain activity and enhances ATP synthesis by reducing superoxide production [23]. Mechanistically, this process involves uncoupling proteins (UCP), which uncouple mitochondrial oxidation (respiration) from ATP production [24].

Furthermore, animal studies have demonstrated that aged mice supplemented with taurine exhibit more efficient glucose metabolism compared to controls; they show decreased blood glucose levels and improved insulin resistance. This metabolic improvement is accompanied by increased cellular autophagy—as indicated by higher LC3A/B content—resulting in the more effective degradation and removal of dysfunctional or foreign cellular components [15,17]. Taurine becomes essential under conditions characterized by increased inflammation, and its protective effects have been documented across various inflammatory pathologies [25]. Recent animal studies have demonstrated taurine’s ability to reduce several inflammatory markers, including interleukin (IL)-1β, IL-6, IL-8, and tumor necrosis factor-alpha (TNF-α) [26]. Concurrently, taurine exerts anti-inflammatory effects by downregulating the production of proinflammatory cytokines in adipocytes [27].

However, the molecular mechanisms by which taurine modulates gene expression related to central metabolic pathways in patients with RA remain insufficiently understood. Therefore, we investigated the effect of taurine on the expression of key metabolic and inflammatory markers in PBMCs and articular cartilage explants from patients with end-stage RA. Specifically, we analyzed pyruvate kinase M2 (PKM2), associated with glycolytic ATP production; succinate dehydrogenase subunit B (SDHB), a component of the Krebs cycle; ATP synthase subunit beta (ATP5B), which is involved in oxidative phosphorylation; and uncoupling protein 2 (UCP2), a marker of mitochondrial activity related to ATP production. We further examined the autophagy-related ULK1, the central metabolic regulator AMPK, and the proinflammatory cytokines TNF-α and IL-1β. Additionally, we analyzed the expression of type II collagen (Col2A1) and matrix metalloproteinase 13 (MMP-13)—both critical components of the extracellular matrix—in knee cartilage explants.

This preliminary study aimed to elucidate how taurine treatment influences genes involved in glycolysis, oxidative phosphorylation, autophagy, and inflammation in these tissues, while also assessing its impact on extracellular matrix gene expression in chondrocytes.

## 2. Materials and Methods

### 2.1. Patients

The study included 20 patients with end-stage rheumatoid arthritis (RA) undergoing arthroplasty, comprising 3 men and 17 women (mean age 62.2 ± 10.9 years). The patients were treated at the Nasonova Research Institute of Rheumatology between June 2024 and June 2025. This study followed the guidelines of the Declaration of Helsinki. The study protocol (No. 12, dated 4 June 2024) was approved by the local ethics committee at the Nasonova Research Institute of Rheumatology, and written informed consent was obtained from all participants. The diagnosis was established in accordance with the 2010 ACR classification criteria.

End-stage rheumatoid arthritis refers to the advanced, severely crippling phase of the disease characterized by extensive joint destruction, deformity, and functional impairment, despite ongoing treatment. It represents the culmination of chronic, uncontrolled RA where joint damage is irreversible and disability is profound. While there is no universally standardized set of criteria, the following features are commonly associated with end-stage RA including primarily, severe joint destruction and deformity such as extensive erosions visible on imaging (X-ray, MRI); fixed joint deformities; loss of joint space and ankylosis. End stage patients with RA also demonstrate multiple joint involvement, namely polyarthritis affecting many small and/or large joints and symmetrical joint destruction. These patients have functional impairment manifested as significant loss of joint function, inability to perform daily activities, and dependence on assistive devices or caregiver support. Persistent disease activity is another trait of end—stage RA which involves chronic, uncontrolled synovitis and resistance to conventional therapies. Radiographic evidence for end stage RA consists of severe joint erosions, osteopenia or osteoporosis around affected joints, and joint space narrowing progressing to ankylosis. In the clinical setting end -stage RA is manifested as chronic pain and swelling, deformities and subluxations, and reduced muscle strength and joint stability.

Inclusion criteria: a confirmed diagnosis of RA (based on the 2010 ACR-EULAR criteria); moderate to high disease activity (DAS28 index > 3.2); age between 20 and 80 years; signed informed consent. Exclusion criteria: pregnancy and lactation; severe active infections (e.g., AIDS, tuberculosis, active viral hepatitis), severe dysfunction of internal organs (renal, hepatic, cardiac failure, uncontrolled hypertension, decompensated diabetes mellitus, etc.), hematological disorders (hemoglobin less than 85 g/L, leukocytes less than 3 × 10^9^/L, platelets less than 100 × 10^9^/L, neutrophils—absolute value less than 2000, lymphocytes—absolute value less than 0.5 × 10^9^/L), elevated liver enzymes (alanine aminotransferase, aspartate aminotransferase above 1.5 times the upper normal limit), or triglycerides above 10 mmol/L; demyelinating diseases of the nervous system; any malignant neoplasms or precancerous conditions, or a history of such within the past 5 years; alcohol or drug addiction. In this preliminary pilot study, we examined a cohort of consecutive patients admitted to the Surgery Department of the Nasonova Research Institute of Rheumatology. All these patients had received various types of medications throughout the course of their disease. Due to the small sample size, it was not possible to establish individual subgroups based on specific previous treatment options. However, in all patients, biological treatments were discontinued three months before surgery. Therefore, the treatment regimens of the examined patients were partially standardized. Table 1 summarizes the key clinical and immunological traits, as well as current and recent treatments, of the examined patients with RA.

### 2.2. Collection and Fractionation of Peripheral Blood Mononuclear Cells

Peripheral blood (10 mL) was collected in EDTA-containing Vacutainer tubes (ZMT, Ekaterinburg, Russia) using a standardized procedure between 07:00 and 09:00. Whole blood was fractionated using a Ficoll density gradient to isolate peripheral blood mononuclear cells (PBMCs). The harvested PBMCs were washed twice with RPMI 1640 medium (Gibco, Life Technologies, Inc., Paisley, UK).

### 2.3. Culture of Peripheral Blood Mononuclear Cells in the Presence of Taurine

PBMCs were seeded in 96-well plates at a density of 5 × 10^5^ cells/mL in RPMI 1640 medium (Gibco, Life Technologies, Inc., Paisley, UK) supplemented with 25 mM HEPES buffer (pH 7.4), 10% (*v*/*v*) heat-inactivated fetal bovine serum (FBS, Gibco, Life Technologies, Inc., Paisley, UK), 100 U/mL penicillin, 100 μg/mL streptomycin, and 150 μg/mL gentamicin sulfate. Cells were incubated at 37 °C in a 5% CO_2_ atmosphere for 24 h. Following medium replacement, cells underwent a 24-h starvation period in the same serum-free medium. Taurine was prepared by dissolving it in water to a stock concentration of 40 mg/mL, which was then diluted with culture medium to a final working concentration of 50 μM. Cells were treated with fresh serum-free medium containing either 50 μM taurine (OTC Pharm, Moscow, Russia) or a vehicle control (medium without taurine). After 1 h of incubation, 10% (*v*/*v*) FBS was added to each well, and cells were incubated for an additional 24 h. Finally, cells were harvested, and total RNA was isolated immediately. All experiments were performed in triplicate.

### 2.4. Viability Test of Peripheral Blood Mononuclear Cells

PBMC viability in patients with RA was assessed 24 h after taurine (OTC Pharm, Moscow, Russia) treatment using 0.2% trypan blue staining, following the same temporal sequence described in Section 2.3. Trypan blue is a vital dye used to evaluate cell viability based on membrane integrity; live cells exclude the dye due to intact membranes, whereas dead or damaged cells allow the dye to penetrate, resulting in blue coloration. For each sample, 2 µL of 0.4% trypan blue solution (Sigma-Aldrich, St. Louis, MO, USA) was mixed with 18 µL of cell suspension (5 × 10^4^ cells/mL) and incubated for 10 min. Samples were examined microscopically using a Leica DM IL LED device (Leica Microsystems CMS GmbH, Wetzlar, Germany), and cell counts were performed using a Goryaev counting chamber. Viability was calculated as the percentage of unstained (viable) cells relative to the total cell count within 10 large squares of the chamber. Ten preparations per experimental group were analyzed, with each preparation containing 50–100 cells per 10 large squares.

### 2.5. Quantification of Protein Levels

Concentrations of SDHB (SEJ756Hu), UCP2 (SEA588Hu), and IL-1β (HEA563Hu) were measured in cultured PBMCs using commercially available ELISA kits (Cloud-Clone Corp, Houston, TX, USA) according to the manufacturer’s instructions. PBMC lysates were prepared using a Cell Extraction Buffer containing 10 mM Tris, pH 7.4, 100 mM NaCl, 1 mM EDTA, 1 mM EGTA, 1 mM NaF, 20 mM Na_4_P_2_O_7_, 20 mM Na_3_VO_4_, 1% Triton X-100, 10% glycerol, 0.1% SDS, and 0.5% deoxycholate (Invitrogen, Camarillo, CA, USA), supplemented with a Protease Inhibitor Cocktail (Sigma-Aldrich, Inc., St. Louis, MO, USA) and 1 mM PMSF (Sigma-Aldrich, Inc., St. Louis, MO, USA), following the manufacturer’s instructions. The total DNA content in PBMC lysates was measured spectrophotometrically using a GeneQuant device (Amersham Biosciences, Cambridge, England). Results were expressed as per microgram of DNA.

### 2.6. Preparation of Articular Cartilage from Patients with RA

Articular cartilage from the distal femur of patients with RA was obtained following total knee arthroplasty. The cartilage was prepared as described previously [27]. Three cubes (20–30 mg each) were pre-cultured in 100 μL of DMEM-A (Dulbecco’s Modified Eagle’s Medium A; Life Technologies) containing 20 mM HEPES buffer, pH 7.4, 45 mM NaHCO_3_, 100 U/mL penicillin, 100 U/mL streptomycin, 150 μg/mL gentamicin sulfate, and ITS (Sigma-Aldrich, Inc., St. Louis, MO, USA). The cartilage cubes were cultured on 96-well Costar 3548 plates for 24 h at 37 °C in an atmosphere of 95% air and 5% CO_2_.

### 2.7. Culture of Cartilage Explants with Taurine Treatment

The medium was replaced after 24 h, marking the beginning of the experiment (Day 0). For the experimental group, DMEM-A medium was supplemented with freshly prepared 50 μM taurine (OTC Pharm, Moscow, Russia) to reach a final concentration of 50 μM. Control samples were maintained in medium without taurine. Cartilage explants (performed in triplicate for both groups) were cultured for an additional 24 h. The knee articular cartilage explants followed the same stimulation schedule as the PBMCs. Upon completion of the experiment, total RNA was extracted from the cartilage samples.

### 2.8. Total RNA Isolation and Reverse Transcription (RT) Reaction

Total RNA was isolated from each well using the Extract RNA reagent (Evrogen, Moscow, Russia). For cartilage explants, an additional step was performed using the Ribosol S kit, according to the manufacturer’s recommendations (InterLabService, Moscow, Russia). The RT reaction was carried out using a kit containing M-MLV reverse transcriptase, random hexanucleotide primers, and total RNA, following the manufacturer’s instructions, with a Reverta kit (InterLabService, Moscow, Russia).

### 2.9. Quantitative Real-Time Polymerase Chain Reaction (qRT-PCR)

Pre-designed primer and probe sets were used for analysis in the TaqMan assay (Applied Biosystems, Foster City, CA, USA): PKM2 (Hs00175407_m1), SDHB (Hs01042482_m1), ATP5B (Hs00969569_m1), AMPKα (Hs01562315_m1), UCP2 (Hs01075227_m1), ULK1 (Hs00177504_m1), TNF*α* (Hs00174128 m1), IL-1*β* (Hs00174097 m1), MMP-13 (Hs00233992 m1), and COL2A1 (Hs00264051 m1). *β*-Actin was used as an endogenous control. Quantification of mRNA levels was performed using a Quant Studio 5 instrument (Applied Biosystems Int., Foster City, CA, USA) as described previously [28]. In the real-time PCR system, the relative expression of each gene was calculated relative to the control, which was set to 1, using the delta-delta Ct (ΔΔCt) method as described by the manufacturer (Applied Biosystems). Relative mRNA expression was calculated using the delta-delta CT method according to the manufacturer’s guidelines. The delta CT value was obtained by subtracting the CT value of the housekeeping gene β-Actin from the CT value of each sample. Then, the delta-delta CT value was determined by subtracting the delta CT of the control cells (without taurine treatment) from that of taurine treated cells from each RA patient.

### 2.10. Statistical Analysis

The normality of data distribution was assessed using the Kolmogorov–Smirnov test. Results are presented as medians with interquartile ranges (25th to 75th percentiles). All analyses were performed in triplicate. Statistical processing was conducted using Statistica for Windows (StatSoft Inc., version 10, Tulsa, OK, USA). Mann-Whitney U tests were used for statistical comparisons. Differences were considered statistically significant at *p* ≤ 0.05. Statistically significant differences compared to the control are marked with an asterisk (*).

## 3. Results

### 3.1. Viability Assay

**Figure 1:**
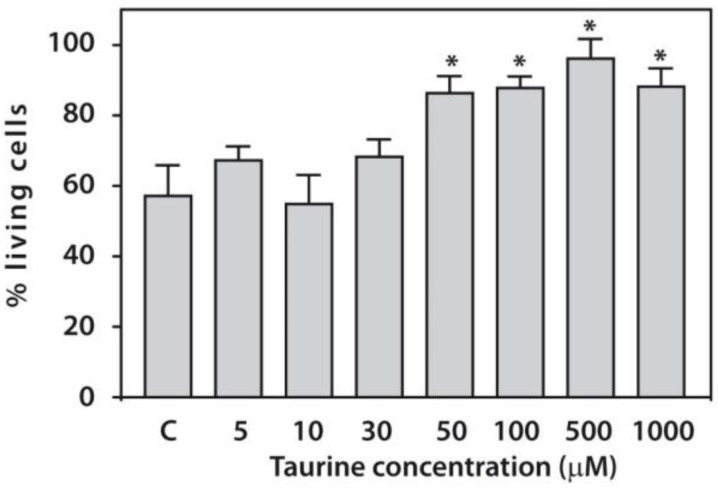
Survivalof PBMCs from patients with end-stage RA (*n* = 20) cultured without (C-control) or in the presence of taurine (5–1000 µM) for 24 h. Asterisks (*) indicate significant differences (Mann–Whitney U-test) in viability of the cells cultured in the presence of taurine compared with control (C).

All tested taurine concentrations (5–1000 μM) were nontoxic, as they did not reduce cell viability compared with untreated cells. Moreover, cell viability increased significantly at taurine concentrations of 50 μM and higher. Therefore, we used 50 μM taurine in all subsequent experiments (Figure 1).

### 3.2. Changes in the Expression of Specific Genes Related to Glycolysis, Oxidative Phosphorylation, Inflammation, and Autophagy in PBMCs Cultured with Taurine from Patients with RA

Our preliminary assessments of gene expression in relation to different taurine concentrations demonstrated that the expression levels of the examined genes, compared to the control, followed the trend of cellular viability (Supplementary Figure S1). Specifically, a significant increase in gene expression was observed starting at a taurine concentration of 50 µM and persisted up to 1000 µM. Additionally, preliminary optimization of PBMC culture incubation times (12, 24, or 48 h) revealed that a significant increase in gene expression compared to control cells occurred after 24 h of incubation (Supplementary Figure S2). Based on these preliminary data, we present gene expression analyses of PBMCs cultured for 24 h with 50 µM taurine.

**Figure 2:**
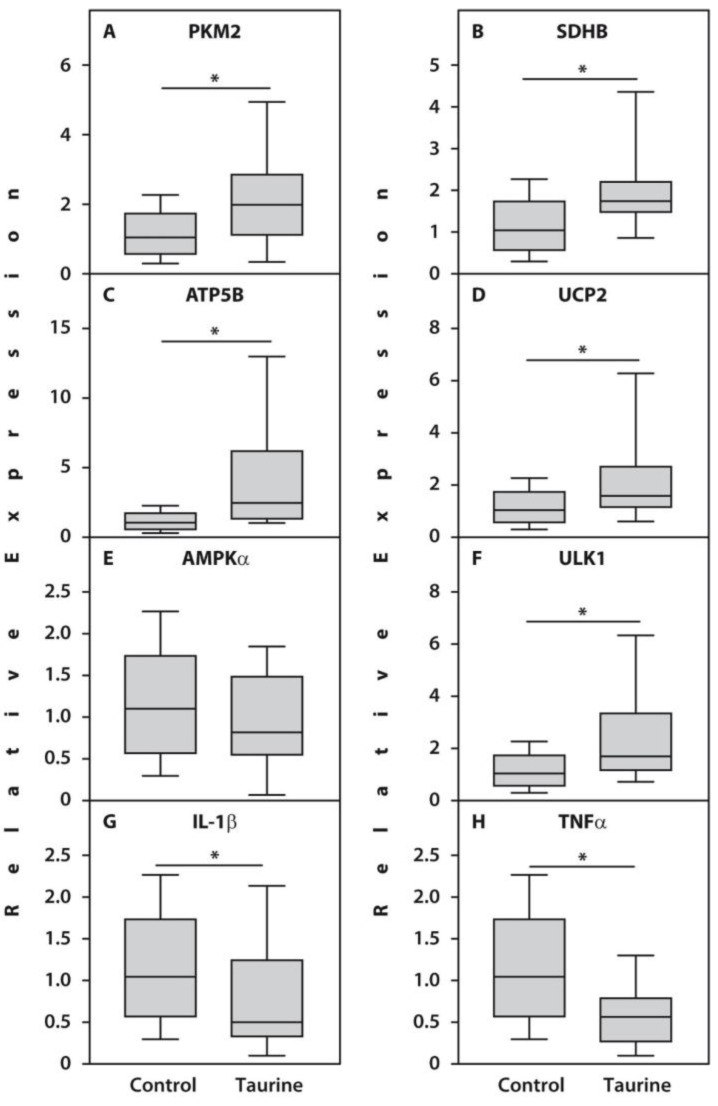
Relative expression of the genes PKM2 (**A**), SDHB (**B**), ATP5B (**C**), UCP2 (**D**), AMPKα (**E**), ULK1 (**F**), IL-1β (**G**), and TNFα (**H**) related to β-actin determined by real-time PCR analyses in the PBMCs treated with 50 µM taurine compared with untreated counterparts of end-stage RA patients (*n* = 20). Controls are shown as 1.0 as required for relative quantification with the real-time PCR protocol. Asterisks (*) indicate significant differences (Mann–Whitney U-test) between examined subsets of cells.

Gene expression analysis of PBMCs cultured with 50 μM taurine revealed a significant increase in the expression of pyruvate kinase, PKM2 (*p* = 0.006), and succinate dehydrogenase, SDHB (*p* = 0.001), compared with untreated controls (Figure 2). Furthermore, a significant increase in ATP synthase expression was accompanied by an increase in the expression of the oxidative phosphorylation uncoupler, UCP2 (*p* = 0.01) and the autophagy marker ULK1 (*p* = 0.003). Conversely, the expression of the proinflammatory cytokines TNFα (*p* = 0.007) and IL-1β (*p* = 0.04) was significantly reduced. The expression of AMPKα did not differ significantly from that in control cells.

### 3.3. Protein Levels of the Examined Genes in Isolated PBMCs

**Figure 3:**
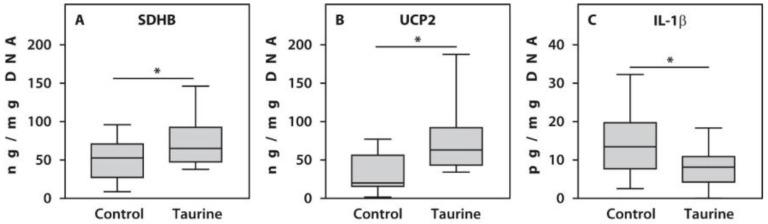
Protein concentrations of SDHB (**A**), UCP2 (**B**), and IL-1β (**C**) measured by ELISA in PBMCs treated with 50 µM taurine compared with untreated counterparts from patients with end-stage RA (*n* = 20). Asterisk (*) indicates significant differences (Mann–Whitney U-test) between examined subsets.

To evaluate the clinical relevance of the relative gene expression levels in the PBMCs of individuals with end-stage RA, we analyzed the protein concentrations of SDHB, UCP2, and IL-1β. Protein levels of SDHB (*p* = 0.04) and UCP2 (*p* < 0.001) in twenty end-stage RA patients treated with 50 μM taurine were significantly higher compared to untreated controls. In contrast, taurine significantly decreased IL-1β (*p* = 0.01) protein levels compared to controls (Figure 3). These results demonstrate that the gene expression data are consistent with the protein expression levels obtained from PBMC samples of the same patients, analyzed using ELISA.

### 3.4. Changes in the Expression of Genes Related to Glycolysis, Oxidative Phosphorylation, Inflammation, Autophagy, and Extracellular Matrix Composition in Articular Cartilage Explants Cultured with Taurine from Patients with RA

**Figure 4:**
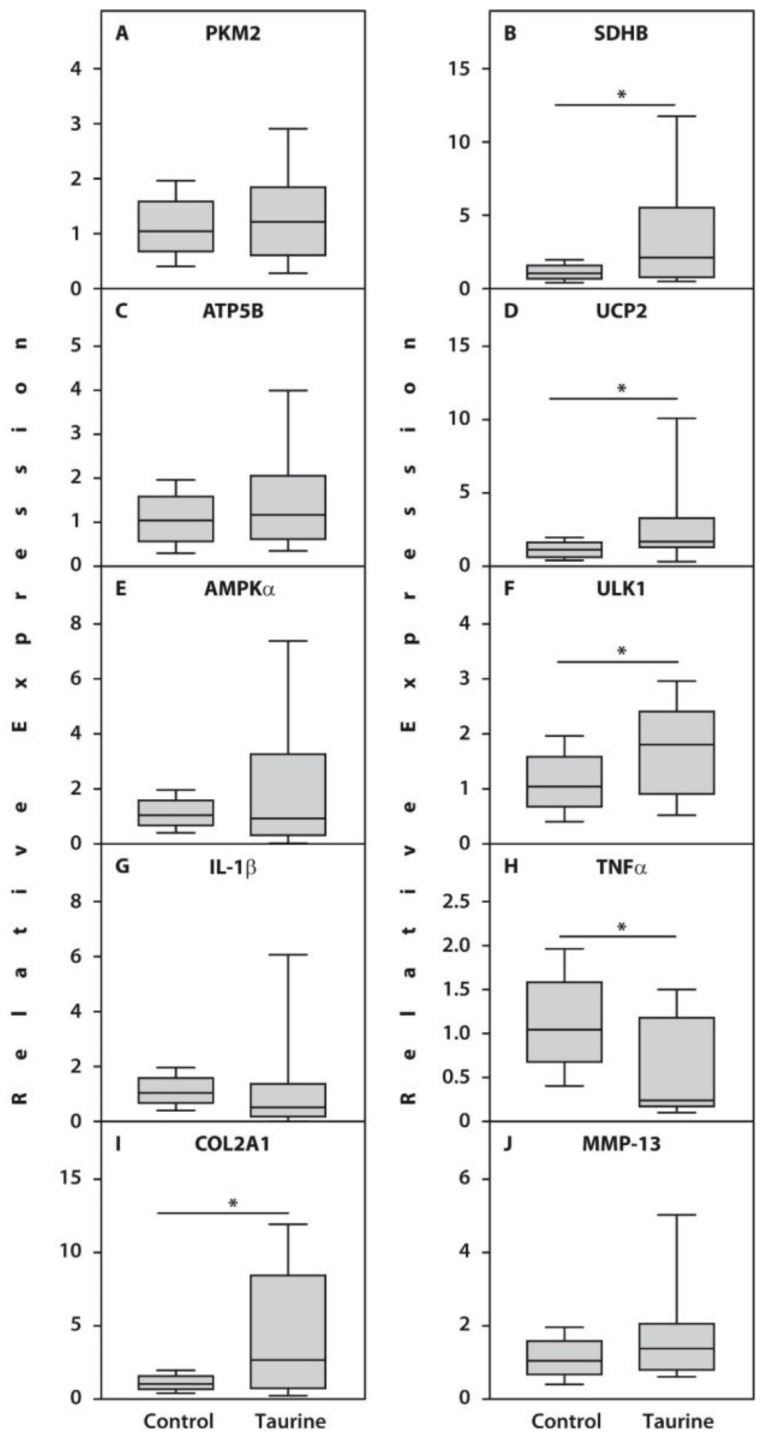
Relative expression of the genes PKM2 (**A**), SDHB (**B**), ATP5B (**C**), UCP2 (**D**), AMPKα (**E**), ULK1 (**F**), IL-1β (**G**), TNFα (**H**), COL2A1 (**I**), and MMP-13 (**J**) related to β-actin determined by real-time PCR analyses in the articular cartilage explants treated with 50 µM taurine compared with untreated counterparts from patients with end-stage RA (*n* = 20). Controls are shown as 1.0 as required for relative quantification with the real-time PCR protocol. Asterisks (*) indicate significant differences (Mann–Whitney U-test) between examined subsets of cells.

Significant changes in the expression of several genes were also observed in articular cartilage explants in response to taurine treatment. Specifically, the presence of 50 µM taurine increased the expression of genes involved in oxidative phosphorylation, such as SDHB (*p* = 0.04), and the uncoupler of oxidation and phosphorylation, UCP2 (*p* = 0.004), while decreasing the expression of TNF-α (*p* = 0.003). Furthermore, a significant increase in type II collagen (*p* = 0.04) expression was observed, indicating activation of its synthesis (Figure 4).

## 4. Discussion

Although taurine influences a wide range of cellular functions—including antioxidant activity, the maintenance of calcium homeostasis, the regulation of cell death, osmoregulation, and the modulation of inflammation—the precise mechanisms underlying these effects remain incompletely understood. Given that most cellular processes rely on the conversion of energy-rich substrates, we investigated the effect of taurine on the expression of genes related to glycolysis, oxidative phosphorylation, inflammation, and autophagy in PBMCs and knee articular cartilage from patients with end-stage RA.

Primarily, we demonstrated that changes in the expression of the examined genes were accompanied by a reduction in the expression of the proinflammatory cytokines TNF-α and IL-1β. Since the overexpression of proinflammatory cytokines is a hallmark of RA [6], this finding is significant. Notably, the downregulation of TNF-α and other proinflammatory cytokines in response to taurine therapy has also been documented in previous studies on colitis [29].

Additionally, we demonstrated that in the presence of taurine, PBMCs from patients with RA exhibited increased expression of the gene encoding pyruvate kinase (PKM2), which is essential for ATP synthesis during glycolysis. Furthermore, we observed a significant upregulation of the gene encoding succinate dehydrogenase (SDHB), which catalyzes the oxidation of succinate to fumarate as part of Complex II of the electron transport chain (ETC) [30]. This finding is consistent with previous studies indicating that taurine deficiency leads to impaired substrate oxidation within the ETC. However, unlike findings in cardiomyocytes—where taurine modulates substrate oxidation solely via Complex I [31]—the activation of the SDHB gene suggests that taurine may also activate Complex II in both PBMCs and cartilage explants from patients with RA. Moreover, the potential benefit of upregulating SDHB and PKM2 in response to taurine is further supported by the increased expression of these genes in patients with RA who achieved remission during tofacitinib therapy [12].

The activation of genes related to the Krebs cycle and the electron transport chain was accompanied by elevated expression of ATP5B, the mitochondrial ATP synthase beta-subunit, in PBMCs. This subunit is involved in ATP synthesis in the inner mitochondrial membrane during oxidative phosphorylation [32]. These findings suggest an improvement in the energy supply of these cells in the presence of taurine, which is particularly significant given the profound energy deficit often observed in the tissues of patients with RA [33]. In this context, our results align with previous observations of a substantial decline in myocardial energy metabolism associated with taurine deficiency [34].

The increased expression of the gene encoding the electron transport chain uncoupler UCP2 in both PBMCs and cartilage explants following taurine supplementation aligns with findings from previous research. For example, studies on isolated cardiomyocytes cultured with β-alanine—a taurine antagonist that inhibits its cellular uptake—have demonstrated an overproduction of superoxide radicals [23]. The upregulation of this uncoupling protein gene suggests a potential restoration of the balance between mitochondrial substrate oxidation and ATP production [23].

Furthermore, we observed a significant increase in the expression of the gene encoding the autophagy marker ULK1 in both PBMCs and articular chondrocytes following taurine treatment. Although we did not directly measure autophagy—a notable limitation of our study—the induction of autophagy-related gene expression may reflect an increased requirement for nutrient recycling and the maintenance of proteostasis [35]. Moreover, while autophagy is known to be activated in RA in a TNFα-dependent manner [36], the upregulation of ULK1 in our study occurred alongside the downregulation of the TNFα gene, suggesting the involvement of distinct molecular mechanisms. Previous animal studies have also demonstrated increased autophagy activity following taurine supplementation [37]. Nevertheless, because our study relied on ULK1 as an indirect marker, its pilot nature and limited sample size necessitate further validation. Future research utilizing more comprehensive autophagy assessments and larger cohorts is essential to confirm and expand upon these preliminary findings.

Regarding the articular cartilage explants, the most significant observation was a marked increase in COL2A1 (type II collagen) gene expression, suggesting a potential for cartilage tissue regeneration even in cases of end-stage RA. While previous animal studies have demonstrated taurine’s ability to inhibit MMP-2 and MMP-9 [38,39], we did not find a corresponding decrease in MMP-13 gene expression. This is notable because MMP-13 serves functions beyond degradation. During development, for instance, MMP-13 is expressed not only in the hypertrophic zone of the growth plate—where cartilage is replaced by bone—but also in the early proliferative zone, where it is essential for chondrocyte proliferation and the formation of extracellular matrix components necessary for bone elongation [40]. Furthermore, animal studies have demonstrated that MMP-13 plays a crucial role in skeletal repair, as it is required for the initial degradation of matrix components during remodeling [41]. Given the severe damage to bone and cartilage networks in RA, the degradation of triple-helical collagens prior to repair may be of particular significance.

It should be noted that taurine supplementation, used in conjunction with conventional anti-rheumatic therapy, could be particularly valuable for managing chronic inflammatory diseases such as RA. Its non-toxic profile allows for long-term administration without the adverse effects that typically necessitate drug discontinuation [42]. Moreover, given that PBMCs and articular chondrocytes from patients with end-stage RA responded to taurine *in vitro*, it is plausible that this intervention may also be beneficial in the earlier stages of the disease. Therefore, further clinical trials exploring the potential role of taurine in RA treatment are warranted.

Currently, as no existing anti-rheumatic drug can cure RA, researchers are exploring novel strategies aimed at achieving sustained, drug-free remission. These emerging approaches range from the development of genetically engineered therapies to the modulation of cellular metabolism and the application of gene-editing techniques [43]. Within this context, dietary interventions—specifically the use of supplements with anti-inflammatory properties, such as taurine—may play a significant role in future RA management strategies [44].

Our findings regarding significant changes in gene expression, supported by protein-level validation following taurine treatment, establish a strong foundation for the extended molecular analyses we intend to pursue. Future studies will broaden this scope to include assessments of cellular metabolite profiles, functional metabolic assays, and post-translational modifications in response to taurine. While our current study focused primarily on gene expression to delineate the molecular mechanisms underlying rheumatoid arthritis pathophysiology, this approach was intentional: it provides a critical baseline for understanding how non-inflammatory processes contribute to disease progression. We believe this gene expression data serves as a necessary starting point, which future multi-omics analyses will complement to offer a more holistic view. Furthermore, expanding this research to include cells and tissues from patients with other rheumatic conditions would be highly valuable.

Furthermore, while in vitro models offer valuable insights into cellular and molecular mechanisms under controlled conditions, they inherently lack the multifaceted interactions present in vivo, such as systemic immune responses, hormonal signaling, vascularization, and crosstalk between diverse cell types and tissues. Although in vitro studies are essential for dissecting specific cellular behaviors, their findings must be interpreted with caution. Consequently, our data should be viewed as an initial mechanistic framework that establishes a foundation for subsequent in vivo and clinical investigations, which are necessary to validate the translational relevance of these observations within the complex environment of a living organism.

While taurine is generally considered safe and well-tolerated, several barriers hinder its widespread adoption as a therapeutic modality. These include the need for further clinical trials to establish optimal dosing, long-term safety profiles, and efficacy across specific patient populations. Furthermore, regulatory hurdles and the requirement for standardized formulations remain significant challenges. Consequently, while taurine supplementation shows promise, these factors must be carefully addressed before it can be recommended as a routine therapeutic intervention.

Regarding the scope of experimental research, it is neither practical nor ethical to test therapeutic agents on cells or tissues derived from healthy individuals. In vitro studies are primarily conducted as precursors to clinical translation; therefore, resources are appropriately prioritized toward understanding a drug’s safety, efficacy, and mechanism of action within the relevant patient cohort. Specifically, in research concerning Rheumatoid Arthritis (RA), investigations typically focus on the patient population for the following reasons: administering experimental drugs to healthy individuals poses significant ethical concerns. It is generally unacceptable to expose healthy volunteers to potential risks or side effects when they cannot derive any direct clinical benefit. The primary goal of such research is to observe how a drug modifies pathological processes unique to RA. Because the biological pathways targeted by the drug may be inactive or regulated differently in healthy individuals, testing on healthy controls could yield negligible, irrelevant, or even misleading data. Many parameters being evaluated—such as disease activity markers or specific inflammatory pathways—are applicable only to the RA population. Consequently, testing these parameters in healthy subjects would fail to provide meaningful data regarding treatment efficacy. Regulatory bodies and ethical guidelines often restrict the testing of investigational drugs on healthy subjects unless the safety profile is exceptionally well-established and a compelling justification exists. Given these considerations, there is little scientific or ethical rationale for investigating the effects of RA-targeted therapeutics on healthy controls.

Our findings provide promising preliminary evidence supporting the potential of taurine as a beneficial supplement for RA management. However, it is essential to conduct further clinical trials to validate these effects in vivo and to fully elucidate the therapeutic potential of taurine in patients with RA.

## 5. Conclusions

Our studies have demonstrated that taurine can alter gene expression in peripheral blood mononuclear cells (PBMCs) and articular cartilage explants from patients with end-stage RA. Specifically, in vitro treatment resulted in the upregulation of genes involved in oxidative phosphorylation (e.g., SDHB) and the initiation of autophagy (e.g., ULK1), alongside the downregulation of inflammation-related markers such as TNFα and IL-1β. Furthermore, taurine was found to upregulate the expression of type II collagen, a key component of the articular cartilage extracellular matrix. Further research is required to explore metabolic responses—specifically regarding metabolite levels and post-translational modifications—in patients with RA following taurine treatment. Such investigations will enhance our understanding of the underlying factors driving rheumatic processes and support the development of innovative therapeutic strategies for improved RA management.

## Figures and Tables

**Table 1:** Clinical and immunological parameters, and treatment of end-stage patients with rheumatoid arthritis (*n* = 20).

Clinical and Immunological Parameters	End-Stage Patients with RA (*n* = 20) (Median [IQR])
Age, years	63.0 [54.5; 72.0]
Disease duration	17.5 [9.7; 25.0]
IgM RF, mU/mL	37.9 [2.6; 115.8]
ACPA, U/mL	36.1 [7.4; 237.5]
CRP, mg/L	4.2 [2.2; 16.5]
DAS 28 (CRP)	4.0 [2.9; 4.5]
Erosion score	6.0 [2.0; 13.0]
Comorbidities	
Type 2 Diabetes	2/20
Obesity	1/20
Hypertension	16/20
Bronchial asthma	3/20
Osteoporosis	7/20
Chronic kidney disease	3/20
Current treatment (in the last 3 months before surgery)	
Corticosteroids	11/20
DMARDs (methotrexate, leflunomide, hydroxychloroquine)	16/20
NSAIDs	1/20
Recent treatment	
Corticosteroids	10/20
DMARDs (methotrexate, leflunomide, hydroxychloroquine)	16/20
Biologics (olokizumab, baricitinib, rituximab, adalimumab, etanercept)	8/20
NSAIDs	3/20

IgM RF, immunoglobulin M rheumatoid factor; ACPA, anti-citrullinated peptide antibody; [IQR], interquartile range.

## Data Availability

The data presented in the study are available on request from the corresponding author.

## Supplementary Materials

The following supporting information can be downloaded at: https://www.mdpi.com/article/10.3390/life16050791/s1, Figure S1: Relative expression of the genes PKM2 (A), SDHB (B), ATP5B (C), UCP2 (D), AMPKα (E), ULK1 (F), IL-1β (G), and TNFα (H) related to β-actin determined by real-time PCR analyses in the PBMCs treated with 5, 10, 30, 50, 100, 500, and 1000 µM taurine compared with untreated counterparts of end-stage RA patients (*n* = 20). Controls (C) are shown as 1.0 as required for relative quantification with the real-time PCR protocol. Asterisks (*) indicate significant differences (Mann–Whitney U-test) between examined subsets of cells. Figure S2: Relative expression of the genes PKM2 (A), SDHB (B), ATP5B (C), UCP2 (D), AMPKα (E), ULK1 (F), IL-1β (G), and TNFα (H) related to β-actin determined by real-time PCR analyses in the PBMCs treated with 50 µM taurine during 12, 24, and 48 h compared with untreated counterparts of end-stage RA patients (*n* = 20). Controls (C) are shown as 1.0 as required for relative quantification with the real-time PCR protocol. Asterisks (*) indicate significant differences (Mann–Whitney U-test) between examined subsets of cells.
