# Supplementary material for: Gene Expression Alterations in Peripheral Blood Mononuclear Cells and Cartilage Explants from End-Stage Rheumatoid Arthritis Patients in Response to Taurine: A Pilot Exploratory Study
Source: Life (Basel). 2026 May 9;16(5):791. doi: 10.3390/life16050791 (PMC13208144; doi:10.3390/life16050791)
Supplement: Supplementary file 1 [file life-16-00791-s001.zip › life-4245698-supplementary.pdf]

Supplementary Figures

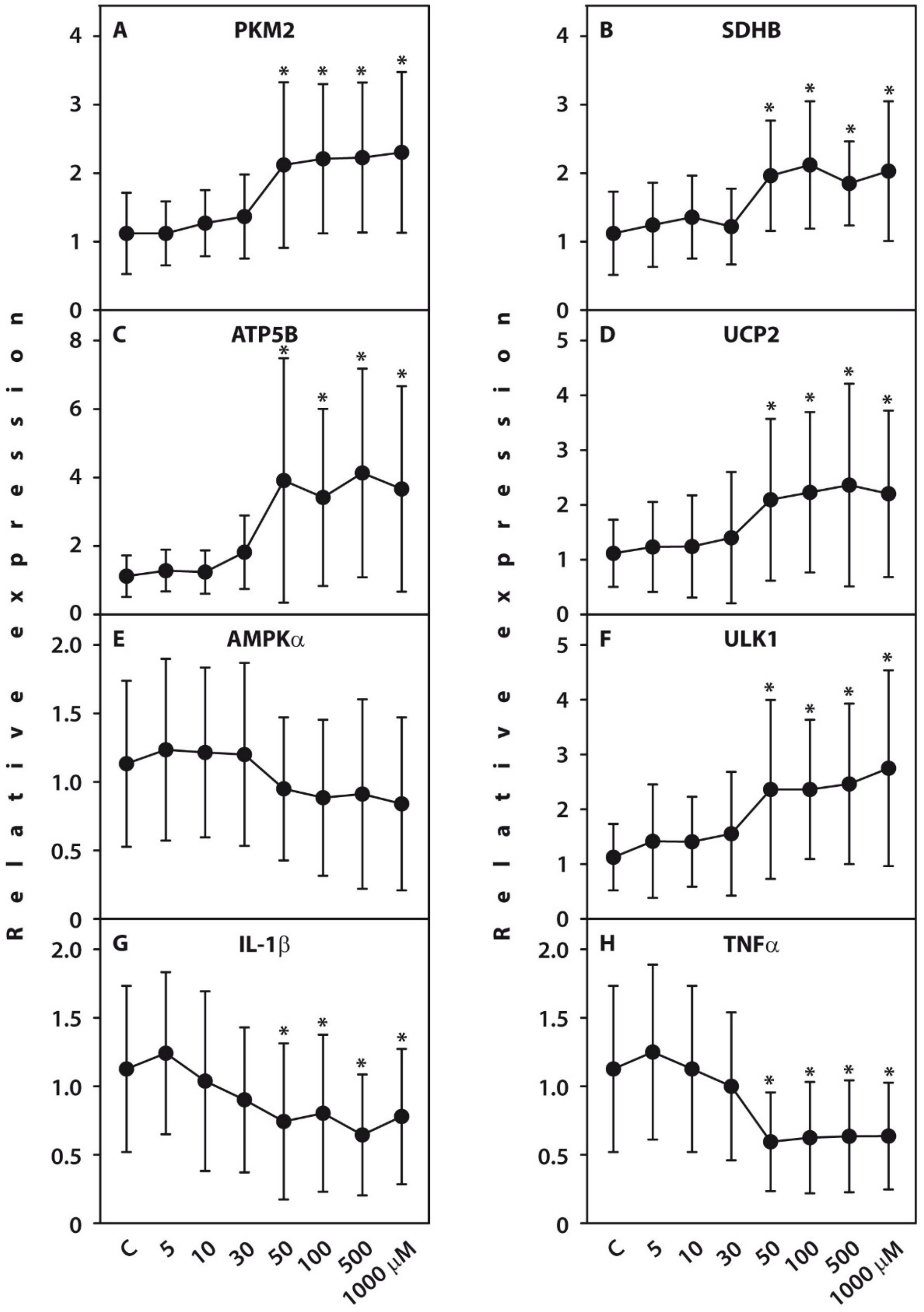

Figure S1. Relative expression of the genes PKM2 (A), SDHB (B), ATP5B (C), UCP2 (D), AMPKα (E), ULK1 (F), IL-1β (G), and TNFα (H) related to β-actin determined by real-time PCR analyses in the PBMCs treated with 5, 10, 30, 50, 100, 500, and 1000μM taurine compared with untreated counterparts of end-stage RA patients (n=20). Controls (C) are shown as 1.0 as required for relative quantification with the real-time PCR protocol. Asterisks (*) indicate significant differences (Mann–Whitney U-test) between examined subsets of cells.

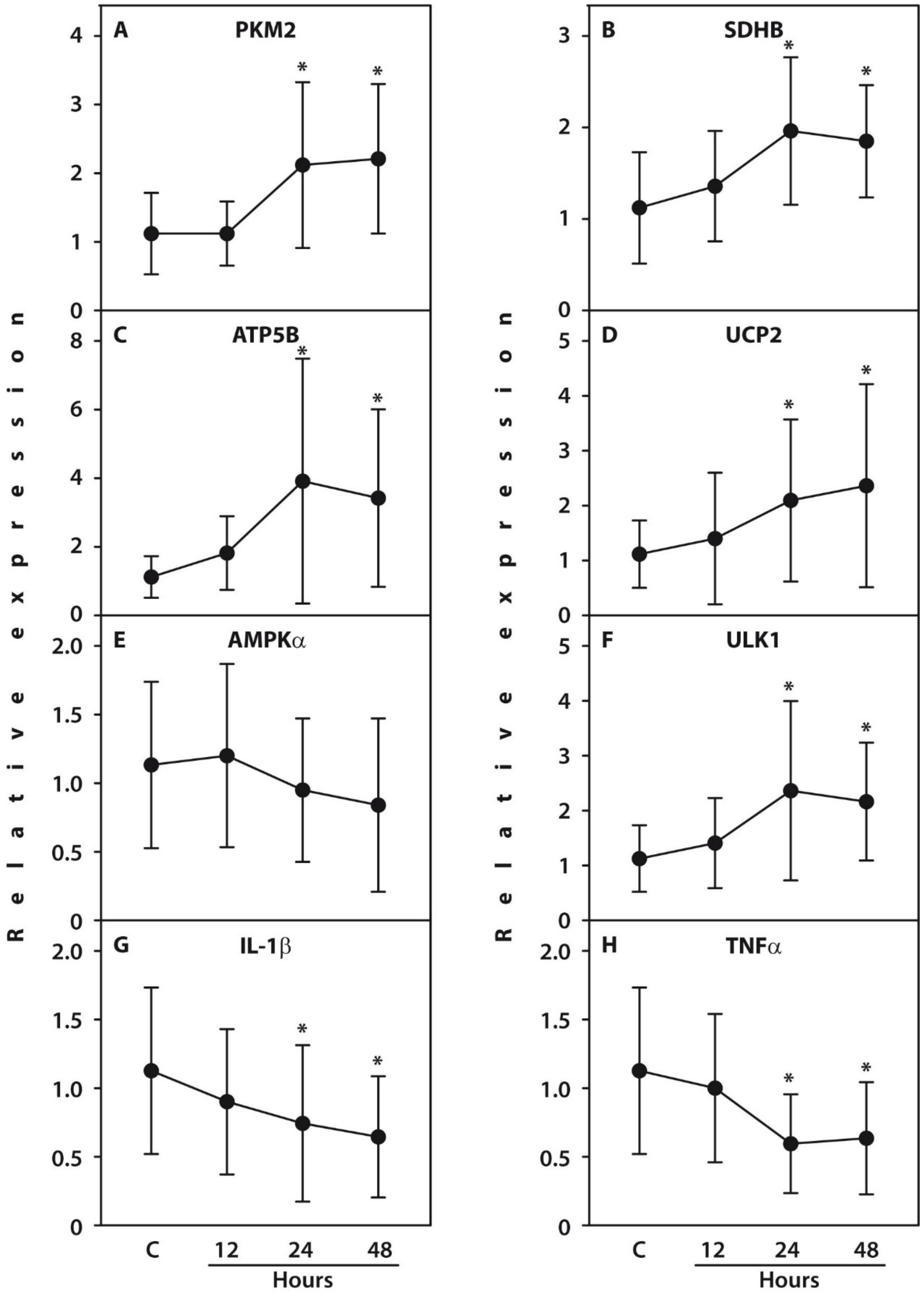

Figure S2. Relative expression of the genes PKM2 (A), SDHB (B), ATP5B (C), UCP2 (D), AMPKα (E), ULK1 (F), IL-1β (G), and TNFα (H) related to β-actin determined by real-time PCR analyses in the PBMCs treated with 50μM taurine during 12, 24, and 48h compared with untreated counterparts of end-stage RA patients (n=20). Controls (C) are shown as 1.0 as required for relative quantification with the real-time PCR protocol. Asterisks (*) indicate significant differences (Mann–Whitney U-test) between examined subsets of cells.
